# Reduction in Dark Current in Photodiodes: A Review

**DOI:** 10.3390/mi17040458

**Published:** 2026-04-08

**Authors:** Alper Ülkü, Ralph Potztal, Tobias Blaettler, Cengiz Tuğsav Küpçü, Reto Besserer, Dietmar Bertsch, Tina Strüning, Samuel Huber

**Affiliations:** 1 ESPROS Photonics AG, 7320 Sargans, Switzerland; 2 OST—Eastern Switzerland University of Applied Sciences, 9471 Buchs, Switzerland

**Keywords:** dark current, photodiode, surface passivation, guard ring, gettering, CMOS image sensor, avalanche photodiode, Shockley–Read–Hall, silicon, germanium, SPAD, ALD passivation

## Abstract

Dark current represents a fundamental limiting factor in photodiode performance, establishing the noise floor and constraining detectivity in low-light applications. This comprehensive literature review examines publications covering the physical mechanisms underlying dark current generation and diverse techniques employed for its reduction. Covered mechanisms include diffusion current, Shockley–Read–Hall (SRH) generation–recombination, trap-assisted tunneling, band-to-band tunneling, and surface leakage, each examined with respect to its physical origin and characteristic signatures. Reduction strategies are categorized into thermal management approaches, surface passivation techniques including atomic-layer-deposited aluminum oxide (ALD Al_2_O_3_), guard ring architectures (attached, floating, and combined configurations), gettering and defect engineering methods, doping profile optimization, bias voltage management, and advanced device architectures such as pinned photodiodes and black silicon structures. A classification table organizes all the reviewed literature by material system, reduction technique, and key findings. Special emphasis is placed on silicon, germanium, III–V compounds, and emerging material photodiodes relevant to near-infrared detection, CMOS imaging, single-photon avalanche diodes (SPADs), and Time-of-Flight (ToF) applications.

## 1. Introduction

Even without incident light, a photodiode produces a small but persistent current—the so-called dark current—driven by thermal carrier generation within the device. This background leakage, which flows regardless of illumination, sets the noise floor of the detector and ultimately limits its minimum detectable optical power, signal-to-noise ratio (SNR), and specific detectivity ($D^{*}$) [1]. Across a remarkably wide range of applications—fiber-optic receivers, medical scanners, LiDAR systems for autonomous vehicles, precision scientific instruments, and quantum information hardware—dark current imposes a fundamental limit on achievable sensitivity, making its suppression a central concern in photodiode engineering [2,3,4].

In practice, dark current is not a single phenomenon but the sum of several physically distinct contributions:

(1) $$J_{\mathrm{dark}}=J_{\mathrm{diff}}+J_{\mathrm{SRH}}+J_{\mathrm{TAT}}+J_{\mathrm{BTBT}}+J_{\mathrm{surf}}$$

Here, $J_{\mathrm{diff}}$ represents the diffusion current attributable to thermally induced minority carriers within the quasi-neutral zones, whereas $J_{\mathrm{SRH}}$ is derived from the Shockley–Read–Hall (SRH) generation mechanism through midgap trap states, $J_{\mathrm{TAT}}$ reflects trap-assisted quantum mechanical tunneling, $J_{\mathrm{BTBT}}$ captures direct band-to-band tunneling, and $J_{\mathrm{surf}}$ accounts for generation at surfaces and interfaces. Which of these terms dominates in a given device depends on a tightly coupled combination of temperature, reverse bias, material crystal quality, device geometry, and operating conditions.

This review is organized around four overlapping goals: first, a grounded account of the physics behind each dark current mechanism; second, a discussion of how temperature and bias measurements can be used to identify which mechanism is dominant in a particular device; third, a survey of practical reduction approaches that span materials science, process engineering, and device design; and fourth, an assessment of recent progress in guard ring geometries, passivation chemistries, and gettering strategies. Material coverage extends across silicon, germanium, III–V compounds (particularly InGaAs/InP), and several emerging platforms including GeSn alloys, blocked impurity band (BIB) detectors, and 2D van der Waals heterostructures, with particular weight given to near-infrared (NIR) detection, CMOS image sensors, single-photon avalanche diodes (SPADs), and Time-of-Flight (ToF) ranging systems [5,6,7]. Narrow-bandgap infrared platforms (HgCdTe, type-II superlattices) are discussed where they provide essential context for dark current mechanisms or benchmarking of emerging alternatives, though a comprehensive review of these mature technologies is beyond the present scope.

The literature surveyed here was selected according to the following criteria: (1) direct relevance to dark current generation mechanisms or reduction techniques in photodiodes; (2) quantitative reporting of dark current, dark count rate, or a directly related metric (with preference for studies providing enough detail to enable cross-comparison); (3) coverage of both mature and emerging material platforms to reflect the current breadth of the field; and (4) inclusion of recent work (2020–2025) alongside foundational references to capture the current trajectory of the field. Where multiple studies report similar findings, preference was given to those providing the most complete device characterization or the most detailed physical analysis of the dark current mechanism involved.

## 2. Fundamental Dark Current Mechanisms

**Figure 1:**
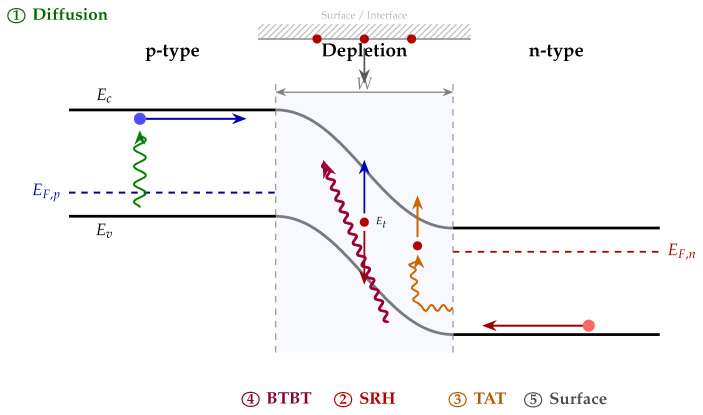
Energy band diagram of a reverse-biased p-n junction illustrating the five principal dark current generation mechanisms: ➀ diffusion of thermally generated minority carriers in the quasi-neutral regions; ➁ Shockley–Read–Hall (SRH) generation through midgap trap states in the depletion region; ➂ trap-assisted tunneling (TAT) via intermediate trap levels under high electric field; ➃ band-to-band tunneling (BTBT) at very high fields; ➄ surface and interface generation through dangling-bond states. Red dots denote trap states; blue and red circles denote electrons and holes, respectively.

Before exploring reduction strategies, it is instructive to examine the physical origins of dark current generation. Each contributing mechanism exhibits a characteristic signature—a distinct temperature dependence, bias sensitivity, and scaling behavior—that both reveals its origin and suggests how it might be controlled. Figure 1 illustrates the five principal dark current generation mechanisms on an energy band diagram of a reverse-biased p-n junction.

### 2.1. Diffusion Current

Thermal excitation in the quasi-neutral regions flanking the depletion zone continually creates minority carriers, which then diffuse toward the junction and are swept across by the built-in field—this constitutes diffusion current [1,8]. Its density follows

(2) $$J_{\mathrm{diff}}=qn_{i}^{2}\left(\frac{D_{n}}{L_{n}N_{A}}+\frac{D_{p}}{L_{p}N_{D}}\right)\left[\exp\left(\frac{qV}{kT}\right)-1\right]$$

Because $n_{i}^{2}\propto \exp\left(-E_{g}/kT\right)$, diffusion current is exquisitely sensitive to temperature: a modest warming can increase it by orders of magnitude. This makes diffusion the dominant mechanism at elevated temperatures in wide-bandgap semiconductors, while narrow-gap materials are often diffusion-limited even near room temperature. In HgCdTe photodiodes, Iakovleva [9] found that diffusion current prevails at very low reverse biases (below approximately 20 mV). For high-quality, defect-sparse silicon photodiodes at room temperature, diffusion current is likewise the leading contributor [10,11].

### 2.2. Shockley–Read–Hall Generation–Recombination Current

All semiconductor crystals contain imperfections: impurities, vacancies, and dislocations introduce localized states within the forbidden gap, and when these trap levels reside near midgap, they become highly effective sites for thermal generation. The Shockley–Read–Hall (SRH) framework [12,13,14] describes how carriers are generated through these states—a process particularly pronounced inside the depletion region, where concentrations are suppressed far below their equilibrium values, making each trap event far more likely to produce a carrier pair than to recombine one. The net generation rate is

(3) $$G_{\mathrm{SRH}}=\frac{n_{i}}{\tau_{g}}=\frac{\sigma_{n}\sigma_{p}v_{\mathrm{th}}N_{t}n_{i}}{\sigma_{n}\exp\left(\frac{E_{t}-E_{i}}{kT}\right)+\sigma_{p}\exp\left(\frac{E_{i}-E_{t}}{kT}\right)}$$

Integrating over the depletion layer gives $J_{\mathrm{SRH}}=qn_{i}W/\tau_{g}$, where *W* is the depletion width. The linear dependence on $n_{i}$ rather than $n_{i}^{2}$ translates into a characteristic thermal activation energy of approximately $E_{g}/2$—a useful diagnostic for separating this mechanism from diffusion in Arrhenius plots [15,16]. Traps situated near midgap are the most effective dark current generators; shallow traps contribute comparatively little [17,18].

### 2.3. Trap-Assisted Tunneling (TAT)

When the electric field in the depletion region becomes significant, carriers need not surmount the full bandgap thermally but can instead tunnel through it with the assistance of intermediate trap states. This trap-assisted tunneling (TAT) process, formalized in the Hurkx model [19,20], produces a current that rises steeply with the electric field:

(4) $$J_{\mathrm{TAT}}\propto N_{t}\exp\left(-\frac{E_{\mathrm{barrier}}}{qF\lambda }\right)$$

where *F* is the local field and λ is a material-dependent tunneling length. In Ge-on-Si photodiodes studied across a wide cryogenic range (34–334 ^∘^K), DiLello et al. [21] showed that fitting activation energies as a function of bias can cleanly separate SRH from TAT contributions. In HgCdTe devices, TAT begins to dominate once the reverse bias exceeds approximately 30 mV [9,22]. In scaled CMOS image sensors, STI edge regions where the field is locally enhanced constitute a significant source of TAT-driven dark current [23,24,25].

### 2.4. Band-to-Band Tunneling (BTBT)

At sufficiently high electric fields, electrons can tunnel directly from the valence band into the conduction band without any trap intermediary—so-called band-to-band tunneling (BTBT) [26]. The Kane model gives

(5) $$J_{\mathrm{BTBT}}=A\frac{F^{2}}{\sqrt{E_{g}}}\exp\left(-\frac{BE_{g}^{3/2}}{F}\right)$$

where *A* and *B* are material constants. The exponential suppression by $E_{g}^{3/2}$ explains why BTBT is substantially more pronounced in narrow-gap semiconductors such as InGaAs, HgCdTe, and GeSn than in silicon [27,28,29]. For mid-infrared detector arrays operating at these wavelengths, cryogenic cooling is not merely helpful but often mandatory: it suppresses BTBT by reducing the thermally assisted component of the field [30].

### 2.5. Surface Leakage Current

Interfaces and exposed surfaces inherently contain defects: dangling bonds, contamination, and lattice strain introduce generation centers that contribute a distinct surface component to dark current [31,32,33,34]. Expressed in terms of a surface recombination velocity $S_{0}$ and the effective generation area $A_{\mathrm{surf}}$,

(6) $$J_{\mathrm{surf}}=qn_{i}S_{0}A_{\mathrm{surf}}$$

This term grows relatively more important as pixel dimensions shrink, since the perimeter-to-area ratio increases and a larger fraction of carriers are generated at or near an interface. In focal-plane arrays with 30 μm or smaller pixel pitches, surface leakage can easily outweigh all other contributions [32,34], underscoring the importance of passivation technologies in image sensor development.

## 3. Temperature-Based Dark Current Reduction

Among all the available approaches, cooling the detector remains the most universally effective approach for dark current reduction. The underlying reason is the exponential sensitivity of every thermally activated generation mechanism to temperature [30,35].

### 3.1. Temperature Dependence Diagnostics

A standard first step in characterizing a new device is to measure dark current over a wide temperature range and extract an Arrhenius activation energy $E_{a}$ [11,36,37,38]. The extracted activation energy provides a diagnostic indicator: $E_{a}\approx E_{g}$ points to diffusion-dominated behavior (current scales as $n_{i}^{2}$), while $E_{a}\approx E_{g}/2$ indicates SRH generation in the depletion region (current scales as $n_{i}$). An apparent activation energy well below $E_{g}/2$ is a strong signal that tunneling—either TAT or BTBT—is playing a significant role [23,24,25].

Systematic measurements on silicon PIN photodiodes spanning 100–400 ^∘^K [38] confirm that dark current can change by many orders of magnitude over this range, while the photocurrent stays nearly flat. The technique of dark current spectroscopy (DCS), which resolves individual defect contributions from their characteristic activation energies and bias dependences, has become a particularly powerful tool for CMOS image sensor development [15,16].

### 3.2. Thermoelectric and Cryogenic Cooling

For applications where detector cooling is practical but liquid cryogens are inconvenient, thermoelectric coolers (TECs) offer a compact solid-state alternative [30]. Even a modest 40 ^∘^K reduction from room temperature provides an approximately adequate improvement in dark current for silicon photodiodes—approximately one order of magnitude per 15 ^∘^K step above 240 ^∘^K [35,39].

Going further to cryogenic temperatures dramatically extends the achievable suppression. In PQED photodiodes, dark current drops from the nanoampere range at room temperature to the picoampere range at 77 ^∘^K [35]—a reduction of several orders of magnitude from a single cooling step. HgCdTe and InSb detectors for the mid-wave infrared routinely require cooling to 100 ^∘^K or below, and operating them uncooled is simply not viable for most applications [30]. For Ge-on-Si devices, measurements across the unusually wide range of 34–334 ^∘^K have proven especially informative: the temperature dependence changes character as different mechanisms become dominant, allowing SRH and TAT contributions to be individually resolved [21]. Separately, controlled cryogenic treatment of silicon wafers prior to device fabrication has also been reported to yield modest but reproducible improvements in detector dark current.

## 4. Surface Passivation Techniques

**Figure 2:**
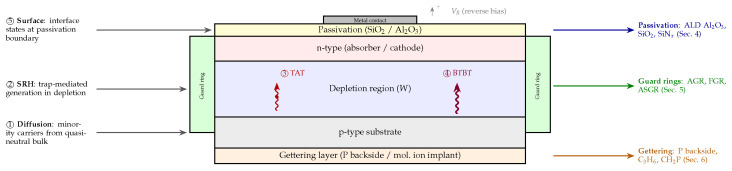
Schematic cross-section of a reverse-biased photodiode showing the spatial origin of each dark current mechanism (left annotations, numbered ➀–➄ corresponding to Figure 1) and the primary reduction strategy that targets each region (right annotations, with section references). Guard rings mitigate peripheral leakage at the junction edges; surface passivation suppresses interface-state generation; gettering layers remove metallic impurities from the active volume. TAT and BTBT occur within the high-field depletion region and are addressed through doping profile and bias optimization (Section 7).

Figure 2 provides a schematic cross-section of a generic reverse-biased photodiode, annotating the spatial origin of each dark current mechanism alongside the reduction strategy that targets it. This device-level view serves as a roadmap for the technique-specific sections that follow.

While cooling addresses all mechanisms simultaneously, surface passivation targets the surface leakage component directly. By reducing the density of interface states and lowering the surface recombination velocity, passivation treatments can cut the surface dark current by multiple orders of magnitude [33,40,41].

### 4.1. Silicon Dioxide and Thermal Oxidation

Thermal SiO_2_ growth on silicon is the historically foundational passivation technique and remains an important reference point [33,42,43]. High-quality thermal oxides achieve interface state densities as low as $10^{10}$ cm^−2^eV^−1^—among the lowest attainable for any semiconductor/insulator combination. In Ge-on-Si photodiodes, post-metallization annealing (PMA) in forming gas after oxide formation can reduce dark current by as much as 1000× by further passivating residual interface states [21]. For photon-trapping silicon nanostructures, the contrast is even more dramatic: properly passivated devices show more than four orders of magnitude lower dark current than unpassivated equivalents [44]. The dominant interface defect at the thermally oxidized Si/SiO_2_ interface is the $P_{b}$ center—an unpaired electron at an Si atom at the interface—and its passivation by hydrogen is well characterized [45,46]. The commercial success of SiO_2_ passivation in solar cells and photodiodes has driven extensive refinement of both thermal and PECVD oxide processes [47].

### 4.2. Atomic-Layer-Deposited Aluminum Oxide (ALD Al_2_O_3_)

Over the past two decades, aluminum oxide deposited by atomic layer deposition (ALD Al_2_O_3_) has evolved from a niche solar-cell technique to an established method for silicon surface passivation. Its effectiveness stems from two complementary mechanisms: excellent chemical passivation of dangling bonds and a large negative fixed charge density ($Q_{f}\approx -10^{13}$ cm^−2^) that creates a field-effect component [40,41,48]. On p-type silicon, this field drives carriers into accumulation; on n-type silicon, it pushes them toward inversion—in either case, minority carriers are shielded from the interface [41,49,50].

The conformal deposition capability of ALD makes it particularly suited to black silicon photodiodes, where conventional planar passivation films fail to coat the deep, high-aspect-ratio nanostructures [51,52,53]. Combining b-Si texturing with ALD Al_2_O_3_ has delivered near-unity quantum efficiency across 250–950 nm [51,54]. More recently, Al_2_O_3_/SiO_2_bilayer stacks with in situ hydrogen plasma treatments reduced the recombination parameter $J_{0}$ to 0.35 fA/cm^2^ [48]—among the lowest values reported for silicon.

Notably, ALD Al_2_O_3_ also outperforms conventional SiO^2^ and SiN_*x*_ films for SiPM and SPAD passivation, where UV sensitivity and low surface generation are jointly required. Emitter saturation current densities as low as 8 fA/cm^2^ have been measured after ALD passivation [55]. The film is thermally stable to approximately 550 °C, so it can be integrated into standard CMOS back-end processes without degradation [43,49].

### 4.3. Silicon Nitride and High-k Dielectrics

Silicon nitride deposited by PECVD or LPCVD carries positive fixed charges, making it a natural complement to Al_2_O_3_ for p-type silicon surfaces and a useful capping layer for passivation stacks [56]. For GaAs-based APDs, comparative studies have shown that SiN_*x*_ passivation achieves markedly lower surface leakage than polyimide alternatives. High-*k* dielectrics including HfO_2_ and Al_2_O_3_ serve dual roles as passivation and gate insulator in detector-transistor integration, with their higher permittivity enabling thicker physical layers for the same equivalent oxide thickness—which reduces tunneling leakage [57]. Non-stoichiometric HfO_*x*_ films have been found to reduce dark current in graphene/Si heterojunction photodetectors while preserving photoresponsivity. Head-to-head comparisons between plasma-assisted ALD and APCVD Al_2_O_3_ suggest both achieve competitive surface passivation, with ALD showing a modest edge in interface defect density [58].

### 4.4. Germanium Surface Passivation

Germanium surface passivation is considerably more difficult than silicon because the Ge/oxide interface has a naturally high density of interface traps that is not easily reduced by simple thermal oxidation [59]. Plasma post-oxidation—exposing the Ge surface to an oxygen plasma to form a controlled GeO_*x*_ layer—reduces the interface trap density below that achievable with deposited SiO_2_/Ge interfaces and cuts dark current by over one order of magnitude [60]. Gas-phase doping combined with GeO_2_passivation takes this further, achieving a bulk current density $J_{\mathrm{bulk}}$ of 0.032 mA/cm^2^ and a surface leakage $J_{\mathrm{surf}}$ of only 0.27 μA/cm [59]—performance competitive with much larger-bandgap materials.

For GeSn photodiodes, the sidewall exposure from mesa etching is an acute problem because the Ge_1−x_Sn_*x*_ surface is even less stable than pure Ge. A thin silicon overgrowth layer conformal to the mesa sidewalls has proven effective at approximately 100× dark current reduction while remaining fully compatible with standard CMOS flows [32,34,61]. This approach circumvents the need for a native GeSn oxide passivation—a problem for which no satisfactory solution has yet been identified. ALD SiO_2_ interlayers at the Ge/Al_2_O_3_ interface allow the effective charge polarity of the passivation to be tuned, opening the possibility of field-effect passivation on Ge analogous to what Al_2_O_3_ provides on silicon.

### 4.5. Limitations and Failure Modes of Passivation Approaches

Despite the impressive reduction figures reported above, several limitations constrain the practical applicability of surface passivation strategies and deserve explicit consideration. Thermal SiO_2_, while providing the lowest interface state densities on silicon, requires processing temperatures of 800–1100 ^∘^C that are incompatible with many back-end-of-line integration flows and with temperature-sensitive materials such as InGaAs or GeSn. ALD Al_2_O_3_ circumvents this with deposition temperatures of 150–300 ^∘^C, but its negative fixed charge—the very property responsible for its field-effect passivation—can induce parasitic inversion channels on n-type surfaces, potentially creating shunt paths that negate the passivation benefit in certain device geometries [40,41]. Furthermore, the long-term stability of ALD passivation under high-fluence UV illumination remains incompletely characterized for space-based applications, where cumulative radiation damage may degrade the Al_2_O_3_/Si interface over mission lifetimes.

For germanium, the fundamental instability of GeO_2_—which is water-soluble and decomposes above approximately 450 ^∘^C—means that any process step exceeding this thermal budget risks undoing the passivation entirely [59]. This places a hard ceiling on the process flows in which Ge passivation can survive, and it is the primary reason why Ge-based detectors have not yet matched the dark current densities of comparably designed silicon devices. The GeSn system faces an even more severe version of this problem: no thermodynamically stable native oxide has been identified for GeSn alloys, and the silicon overgrowth passivation technique, while effective, adds epitaxial complexity and may introduce strain-related defects at high Sn fractions where the lattice mismatch is largest [32,34].

More broadly, the passivation results reported in the literature are often measured on dedicated test structures under optimized conditions. Transferring these results to full detector arrays—where passivation must survive dicing, wire bonding, packaging, and years of field operation—typically yields more modest improvements than the headline figures suggest. The gap between laboratory passivation quality and production-line reliability remains a significant, though infrequently quantified, bottleneck.

## 5. Guard Ring Structures

**Figure 3:**
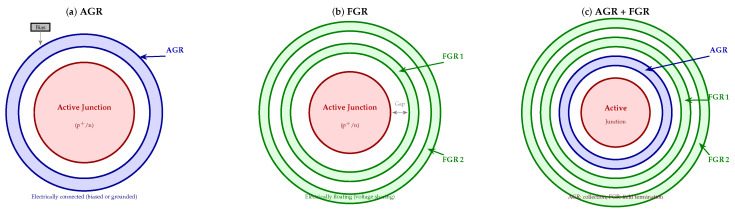
Top view of the three principal guard ring configurations surrounding a circular active junction. (**a**) Attached guard ring (AGR): a diffused ring electrically connected to the active junction or to a bias pad, which intercepts peripherally generated carriers. (**b**) Floating guard ring (FGR): one or more electrically isolated diffused rings separated by gaps from the active area; as the depletion region expands, each ring charges capacitively and shares part of the applied voltage, reducing the peak electric field at the junction edge. (**c**) Combined AGR + FGR: the AGR handles inner peripheral carrier collection, while outer FGRs provide field termination, achieving superior dark current suppression and breakdown voltage improvement compared with either structure alone.

**Figure 4:**
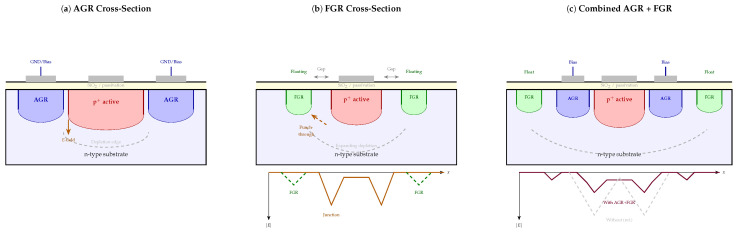
Cross-sectional view of the three guard ring configurations with representative electric field profiles. (**a**) Attached guard ring (AGR): the AGR diffusion is electrically connected via a metal contact and biased (or grounded) to actively collect peripheral carriers. (**b**) Floating guard ring (FGR): isolated diffused regions separated from the active junction by a gap; the expanding depletion region punches through to each ring, which then shares part of the applied voltage. The electric field profile (bottom) shows that voltage sharing at the FGR locations reduces the peak field at the main junction edge. (**c**) Combined AGR + FGR: the AGR is placed immediately adjacent to the active junction for peripheral carrier collection, while outer FGRs provide additional field termination. The combined electric field profile (purple) is substantially lower than the unguarded reference (gray dashed), illustrating the benefit of addressing both inner and outer field regions simultaneously.

Planar junctions exhibit an inherent geometric vulnerability at their edges: the curvature of the diffusion profile locally concentrates the electric field well beyond the value it takes in the flat central region. Without some form of corrective structure, this peripheral field enhancement triggers breakdown and generates leakage long before the intended operating voltage is reached. Guard ring structures were developed precisely to address this problem, redistributing the electric field in a controlled way [62,63,64]. Figure 3 shows a top view of the three principal guard ring configurations, while Figure 4 presents the corresponding cross-sectional views together with representative electric field profiles.

### 5.1. Attached Guard Ring (AGR)

The attached guard ring (AGR) is a diffused region that surrounds the active junction and is electrically connected—typically tied to ground or a separate bias potential [62,64]. Its primary role is to intercept surface-generated carriers before they reach the active area, thereby acting as a current drain rather than allowing the peripherally generated charge to contribute to the noise. TCAD simulations show that the optimal AGR diffusion depth is not simply as deep as possible but rather corresponds to the point at which the breakdown locus transfers from the active junction edge into the AGR itself—a crossover that can be identified cleanly in simulated *I*–*V* curves [64].

Operating an AGR at a modest forward bias of approximately 0.8 V actively drives peripheral collection, achieving dark current densities around 100 nA/cm^2^ [62,65]. Optimization of the AGR depth—targeting 0.2–0.3 μm shallower than the main junction—yields reported improvements of 70% in dark current and 43% in quantum efficiency simultaneously [62]. Double-diffused planar InGaAs/InP APDs are among the structures where AGR design has been most carefully studied because edge breakdown in these devices is an especially serious failure mode [64,66].

### 5.2. Floating Guard Ring (FGR)

Where the AGR actively collects peripheral carriers, the floating guard ring (FGR) works passively by voltage sharing. Each FGR is an electrically isolated diffused ring; when the expanding space-charge region punches through to it, the ring charges up capacitively and redistributes part of the voltage drop [63,67]. The net effect is a gentler, more gradual potential gradient at the junction edge and a reduction in peak electric field [63,68].

In practice, a single FGR can cut peripheral dark current by approximately an order of magnitude [62,69], but placement is critical. The ring must be spaced far enough from the main junction—typically 3–5 μm in InGaAs/InP structures—to achieve breakdown voltage gains on the order of 1.5 V; too close and the punch-through voltage increment becomes negligible [64,70]. The ring width (2–10 μm) and diffusion depth are also consequential design variables [64,71].

For high-voltage applications requiring multiple FGRs, the necessary number can be estimated from

(7) $$N_{\mathrm{FGR}}\geq \frac{V_{\max}-V_{\mathrm{br},1}}{V_{\mathrm{punch}}}$$

where $V_{\mathrm{br},1}$ is the single-junction breakdown voltage and $V_{\mathrm{punch}}$ is the punch-through voltage increment per ring. Multi-ring designs with optimized non-uniform spacing have demonstrated aggregate breakdown voltage increases exceeding 38 V [70,72].

### 5.3. Combined and Advanced Guard Ring Strategies

AGRs and FGRs address complementary failure modes—the AGR handles inner peripheral collection, while the FGR manages outer field termination—so combining them naturally outperforms either structure alone [62,64,64]. Well-optimized combined designs have achieved 50–70% reductions in peripheral dark current alongside up to 90% quantum efficiency improvements, a combination that would be difficult to reach through either approach independently [62,73].

Adding n^+^- and p^+^-guard regions together further restricts leakage channels [74,75], and multi-stage gradient ring geometries—where the spacing or doping is intentionally graded—provide smoother field profiles that suppress premature breakdown more completely [76]. For high-density focal plane arrays, where conventional guard rings consume too much pixel area, guard hole configurations offer an architecturally different solution, reducing dark current density by around 60% with a smaller area penalty.

Among more recent innovations, attached step guard rings (ASGRs) have demonstrated a 23% increase in breakdown voltage relative to conventional AGR structures, along with a three-fold gain increase and more than 20% improvement in quantum efficiency [77]. At the other extreme, guard-ring-free designs using selective area Zn diffusion for InGaAs/InP devices have achieved a 33–43% photon detection probability with dark count rates of 430 cps/μm^2^ at room temperature [78]—suggesting a potential approach that avoids peripheral leakage entirely.

### 5.4. Scaling Bottlenecks and Design Trade-Offs in Guard Rings

Guard ring effectiveness is subject to several practical constraints that are not always apparent from the reported dark current or breakdown voltage improvements. The most fundamental is an area trade-off: each guard ring consumes a lateral chip area that cannot be used for photon collection, and in high-density focal plane arrays, this area penalty directly reduces the fill factor. For small-pitch pixels (<10 μm), even a single floating guard ring can consume a significant fraction of the pixel footprint, motivating the search for area-efficient alternatives such as guard hole geometries. The optimal guard ring parameters—ring width, spacing, depth, and number—interact in a multi-dimensional design space that is difficult to navigate without TCAD simulation, and the optima are highly sensitive to the specific doping profile and substrate resistivity of the target process [62,63,64].

A second limitation is that most guard ring optimization studies are performed on discrete test diodes or small arrays, and the reported improvements may not transfer directly to production-scale focal plane arrays where process uniformity, lithographic registration tolerances, and wafer-level doping variations all degrade the idealized performance. In InGaAs/InP devices, where guard ring design is especially consequential, the diffusion profiles are controlled by zinc diffusion—a process with inherently less spatial control than ion implantation—making reproducible fabrication of optimized guard ring geometries a persistent manufacturing challenge [79]. Additionally, the voltage-sharing mechanism of floating guard rings is inherently sensitive to surface charge and passivation quality; if the overlying dielectric accumulates charge during operation or radiation exposure, the punch-through voltage per ring shifts, potentially invalidating the original design optimization.

## 6. Gettering and Defect Engineering

**Figure 5:**
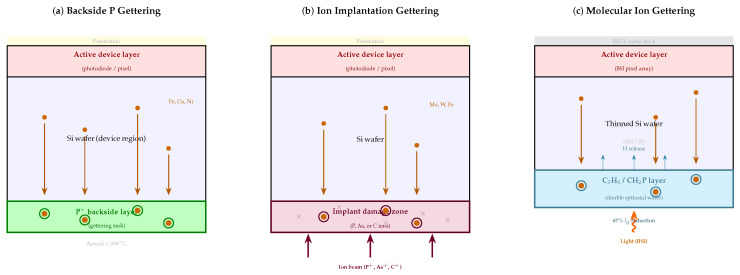
Schematic comparison of three gettering approaches for removing metallic impurities from the photodiode active region. Orange dots represent dissolved metallic impurities (Fe, Cu, Ni, Mo, W); orange arrows indicate thermally driven impurity migration toward the gettering sink; circled dots denote trapped (gettered) impurities. (**a**) Backside phosphorus gettering: a heavily doped P^+^ layer at the wafer backside provides a segregation sink during thermal annealing below 900 ^∘^C. (**b**) Ion implantation gettering: implanted species (P, As, or C) create a localized damage zone that precipitates metallic impurities; carbon implantation is particularly effective against Mo and W. (**c**) Hydrocarbon molecular ion gettering for back-side-illuminated (BSI) CMOS image sensors: C_3_H_6_ or CH_2_P molecular ions implanted into a double epitaxial wafer provide simultaneous metallic impurity gettering, hydrogen release for interface passivation, and an oxygen diffusion barrier—achieving up to 67% dark current reduction [75,81].

Even in well-controlled fabrication environments, metallic impurities—iron, copper, nickel, and others—are incorporated into silicon wafers and create deep trap levels that dominate SRH generation. Gettering strategies exploit the thermodynamic tendency of these impurities to segregate toward specially created high-energy sites, drawing them away from the device active region [74,75,80]. Figure 5 illustrates the three principal gettering approaches—backside phosphorus gettering, ion implantation gettering, and hydrocarbon molecular ion gettering—alongside the impurity migration mechanism common to all three.

### 6.1. Phosphorus and Ion Implantation Gettering

Phosphorus gettering via a heavily doped backside layer is one of the oldest and most widely used techniques [74,80,82]. The high phosphorus concentration provides a substantial gettering driving force for mobile metal impurities. A key practical constraint is temperature: processing above 900 °C can reduce minority carrier lifetime in the device region, so gettering steps are generally kept below this threshold [74,83].

Ion implantation offers a complementary route. Phosphorus implantation at 10^16^ cm^−2^ and 50 keV creates a local damage zone that acts as an efficient gettering sink and has been shown to completely eliminate white video defects. Arsenic at doses of ≥10^15^ cm^−2^ is similarly effective at precipitating metallic impurities [84,85]. More recently, carbon implantation has attracted interest specifically because it captures Mo and W, contaminants that have grown more problematic as advanced CMOS image sensors have encountered these elements from chamber hardware and metallization [86].

### 6.2. Bulk Micro Defects (BMDs) as Internal Gettering Sites

A closely related mechanism exploits bulk micro defects (BMDs)—oxygen precipitates that form naturally in Czochralski-grown silicon during controlled thermal processing. When interstitial oxygen, present at concentrations of $∼10^{17}$–$10^{18}$ cm^−3^ in CZ wafers, is subjected to a multi-step thermal cycle (typically nucleation at 600–800 ^∘^C followed by growth at 1000–1100 ^∘^C), it precipitates into SiO_*x*_ clusters that create strain fields and associated defect complexes in the wafer bulk [75,80]. These BMD sites act as internal gettering sinks: dissolved metallic impurities preferentially segregate toward the strained regions surrounding each precipitate, effectively sweeping the device-active near-surface zone (the stripped zone) clean of electrically active contaminants.

The effectiveness of BMD-based internal gettering depends on several parameters: the initial oxygen concentration (which determines the maximum achievable BMD density), the nucleation and growth thermal profiles (which control precipitate size and spatial distribution), and the denuded zone depth (which must be large enough to encompass the entire active device region). For modern CMOS image sensors, a denuded zone depth of 10–50 μm is typically specified [75,80]. BMD densities in the range of $10^{8}$–$10^{10}$ cm^−3^ provide sufficient gettering capacity for most contaminant levels encountered in production environments. A key advantage of BMD gettering over backside-only techniques is that the gettering sinks are distributed throughout the wafer bulk, reducing the diffusion distance that impurities must travel and thus improving the capture efficiency for slow-diffusing species. However, if BMDs form within the denuded zone itself—due to inadequate thermal profile control—they act as generation centers rather than gettering sinks, directly increasing dark current. This dual role makes precise thermal process control essential for BMD-based gettering strategies.

### 6.3. Hydrocarbon Molecular Ion Gettering for CMOS Image Sensors

Modern back-side-illuminated CMOS image sensors present a particular challenge for gettering because the wafers must be thinned aggressively, eliminating the conventional backside gettering layer. Hydrocarbon molecular ion implantation was developed as a response to this constraint [74,75,81,87]. Implanting C_3_H_6_ into double epitaxial wafers achieves a 40% reduction in white spot defects [87], and substituting CH_2_P molecular ions yields a 67% reduction in dark current—a substantial gain from what is essentially a substrate engineering step [81].

The effectiveness of these hydrocarbon molecular ion layers is attributed to a combination of three properties: metallic impurity gettering capability, localized hydrogen release that passivates nearby interface defects, and an oxygen diffusion barrier effect that prevents oxygen from migrating into the device region during subsequent thermal processing [75,87]. A critical structural factor is that defects formed in double epitaxial layers have unusually low internal oxygen concentration, which enhances their gettering capacity compared to conventional bulk silicon sinks [87].

### 6.4. Defect Density Impact and Threading Dislocation Reduction

The relationship between structural defects and dark current is most clearly illustrated in GeSn p-i-n photodiodes, where the Sn incorporation introduces threading dislocations at densities that vary strongly with composition. Detailed analysis [88] shows that at low Sn fractions, the dark current budget is dominated by defect-related SRH generation, whereas at higher Sn concentrations the bulk thermal generation current becomes competitive. This compositional crossover is a useful design consideration when targeting specific detection wavelengths.

Threading dislocation density reduction through epitaxial growth optimization is consequently a first-order priority for all Ge-based detectors [21,89,90]. Cyclic thermal annealing—repeatedly cycling through temperatures around 850 °C—has been shown to reduce threading dislocation density in Ge-on-Si films substantially by driving dislocation-dislocation annihilation reactions [89]. Ge vertical p-i-n structures fabricated on oxygen-annealed substrates, where oxygen trapping further limits dislocation motion, achieve a particularly low dark current [91,92].

### 6.5. Limitations of Gettering and Defect Engineering

Gettering is most effective against mobile metallic impurities (Fe, Cu, Ni) that can diffuse to the gettering sink within practical annealing times. Slow diffusers—including Mo and W from chamber hardware—require either higher temperatures or longer process times that may conflict with thermal budget constraints imposed by other device layers [75,86]. Furthermore, gettering is fundamentally a bulk purification strategy: it does not address interface-generated dark current or structural defects such as threading dislocations, which must be mitigated by separate means. This distinction is especially important for Ge-on-Si and GeSn detectors, where the dominant dark current contribution often originates from epitaxial defects rather than from dissolved metallic impurities.

For modern back-side-illuminated CMOS image sensors, the elimination of the backside gettering layer during wafer thinning creates a dilemma that hydrocarbon molecular ion implantation only partially resolves. The gettering layer must be placed close enough to the device region to capture impurities before they reach the active volume but far enough away to avoid introducing additional defect-related generation centers. The reported 40–67% reductions in dark current or white spot defects [81,87], while substantial, still leave a residual tail of hot pixels that is attributable to impurities not captured by the gettering sink. In 3D-stacked sensor architectures, where the thermal budget is further constrained by copper interconnect integrity, the temperature window available for effective gettering narrows considerably, and it remains unclear whether current hydrocarbon molecular ion techniques can maintain their effectiveness under these tighter constraints.

In Ge-based systems, cyclic thermal annealing reduces threading dislocation density but cannot eliminate it entirely—a residual density on the order of $10^{6}$–$10^{7}$ cm^−2^ typically persists even after optimized annealing sequences [89,90]. This residual defect density sets a material-quality floor on the achievable dark current that no subsequent process step can overcome, and it explains why Ge-on-Si photodiodes have not yet reached the dark current levels predicted by bulk material parameters alone.

## 7. Device Design and Architecture Optimization

Beyond process and passivation choices, the geometric and electrical design of the device itself has a substantial influence on dark current. The sections below address the key design degrees of freedom.

### 7.1. Doping Profile Optimization

Device-level choices about doping profiles and layer thicknesses have a direct bearing on dark current, independently of material quality or passivation [2,93,94]. A lower doping in the intrinsic region reduces the peak electric field—helpful for suppressing TAT and BTBT—but simultaneously widens the depletion layer and thus increases the SRH generation volume. This trade-off has no universal optimum; it must be resolved against the specific operating voltage and target noise floor of a given application. In InGaAs/InP APDs, the donor concentration in the multiplication layer determines whether breakdown is gain-limited or generation–recombination-limited [95], and only within a specific thickness window does the device achieve the desirable regime where SRH generation, rather than tunneling, governs the dark current [96].

For UV-sensitive and short-wavelength applications, minimizing implant damage near the surface is essential because photons are absorbed within the first few nanometers of the junction. Plasma doping and gas-phase doping techniques allow ultra-shallow junction formation with far less crystal damage than conventional ion implantation [59].

### 7.2. Heterojunction and Thin Absorber Designs

Band engineering through heterojunction design offers a different route: by confining the absorber and controlling carrier injection at the interface, dark current can be suppressed without sacrificing optical performance [30,97]. A lateral heterojunction design on black silicon, for example, reduced dark current to 783 nA at −5 V by moving the generation-prone junction away from the nanostructured surface [97].

InGaAs/Si heterojunction APDs realized by wafer bonding achieve high multiplication gain with a notably weak temperature coefficient, a practical advantage for field-deployed systems that experience wide temperature excursions [98]. Among 2D materials, both MoS_2_ and VS_2_ heterostructures have yielded ultra-low dark currents that reflect the atomically sharp interfaces and reduced bulk defect volume inherent to van der Waals assembly [99,100].

### 7.3. Pinned Photodiode Architecture

The pinned photodiode (PPD), introduced by Teranishi in 1982 and since adopted in essentially all modern CMOS image sensors, achieves an inherently low dark current by architectural design rather than relying solely on passivation chemistry [6,101]. A heavily doped p^+^ layer at the silicon surface pins the potential and fully depletes the underlying n-type storage region, eliminating the Si/SiO_2_ interface as a generation source [5,6]. Because the surface—which would otherwise be the dominant dark current source in a conventional photodiode—is no longer part of the active generation volume, PPD pixels achieve dark current levels that are difficult to match with surface-exposed structures.

Under radiation exposure, hole collection pinned photodiodes show less dark current degradation than conventional electron-collection designs, making them the preferred choice for space and high-radiation environments. Optimizing the transfer gate—including operating it under a small negative bias—further suppresses the residual dark current contribution from the STI and gate oxide interfaces that adjoin the transfer channel [102].

### 7.4. Black Silicon Photodiodes

Black silicon surfaces—where dry or wet etching has created dense arrays of sub-wavelength pillars or cones—reflect less than 1% of incident light across the visible and near-UV, but this optical advantage comes with a surface area that would generate unacceptable dark current without careful passivation [51,52,54]. ALD Al_2_O_3_ is the enabling passivation layer here: its conformal deposition coats even high-aspect-ratio structures uniformly, and its field-effect passivation is robust against the geometric complexity of the nanostructured surface [51,53,103]. Induced-junction b-Si photodiodes formed by the negative charge of the Al_2_O_3_ film rather than by implantation achieve external quantum efficiency above 96% across 250–950 nm [51].

A notable result in this area is that b-Si UV photodiodes can exceed 100% external quantum efficiency due to impact ionization-driven carrier multiplication within the nanostructures—a phenomenon that appears to require both the light-trapping geometry and the low-defect interface provided by ALD passivation [104,105]. Self-biased b-Si heterojunction photodiodes using nanocrystal ITO contacts achieve broadband near-100% quantum efficiency without any external power supply [106], and b-Si devices have been demonstrated with measurable photoresponse extending into the vacuum ultraviolet at 175 nm [104].

### 7.5. Blocked Impurity Band (BIB) Photodetectors for VLWIR

**Figure 6:**
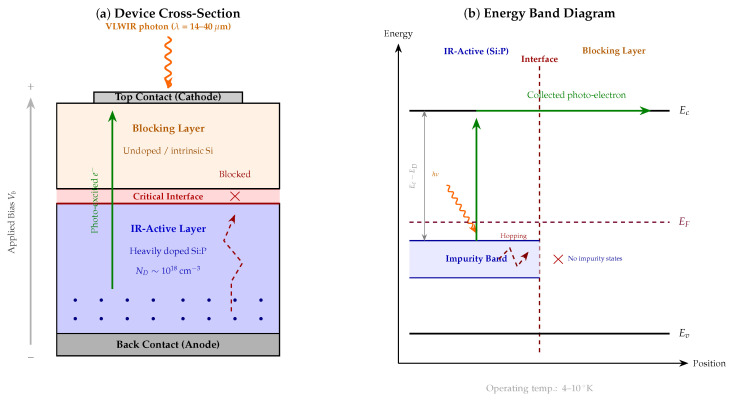
Blocked impurity band (BIB) photodetector for VLWIR detection. (**a**) Device cross-section showing the heavily doped Si:P infrared-active layer, the critical interface, and the undoped blocking layer. Photo-excited electrons (green arrow) are promoted from the impurity band to the conduction band and collected through the blocking layer, while thermally driven hopping conduction (red dashed path) is blocked at the interface. (**b**) Corresponding energy band diagram illustrating the impurity band within the Si bandgap, the sub-bandgap photon absorption process (hν), and the absence of impurity states in the blocking layer that prevents hopping transport. A sharp epitaxial interface suppresses dark current by up to five orders of magnitude relative to a gradual interface [107].

Blocked impurity band (BIB) photodetectors offer a distinct approach to very-long-wavelength infrared (VLWIR, 14–40 μm) detection by exploiting impurity-level transitions in heavily doped silicon rather than intrinsic band-to-band absorption. In a BIB structure, a thin undoped blocking layer suppresses hopping conduction through the impurity band so that only photo-excited carriers are collected, while thermally generated hopping current—which would otherwise dominate at these long wavelengths—is strongly attenuated [107,108]. Figure 6 illustrates the device architecture and the corresponding energy band structure that enables selective carrier collection.

Interface engineering between the doped infrared-active layer and the blocking layer is a first-order determinant of dark current in these devices. A recent interface model for phosphorus-doped silicon (Si:P) BIB detectors has demonstrated that a sharp epitaxial interface produces a well-defined potential barrier that suppresses dark current by five orders of magnitude relative to a gradual interface, maintaining dark current below 1 pA at forward bias up to 2.7 V and achieving a peak blackbody detectivity exceeding $1\times 10^{12}$ cm Hz^1/2^ W^−1^ at 28.3 μm [107]. This result underscores that, even in an architecture specifically designed around impurity transitions, interface quality remains the controlling factor for dark current—a theme consistent with the passivation-dominated behavior observed in conventional photodiodes.

At the focal plane array level, a 16 × 16 Si:P BIB prototype FPA operating at 4 ^∘^K has demonstrated broadband VLWIR detection with a 50% cutoff wavelength of 32.2 μm, a peak detectivity of $∼1\times 10^{11}$ cm Hz^1/2^ W^−1^, and dark current below 40 fA per pixel [108]. The readout integrated circuit was co-designed for deep cryogenic operation, highlighting that system-level dark current management in BIB FPAs requires joint optimization of detector and circuit. These results position Si:P BIB technology as a viable platform for future space-based and ground-based broadband VLWIR imaging where ultra-low dark current under cryogenic conditions is essential.

The principal limitations of BIB detectors are the mandatory deep cryogenic cooling requirement (typically 4–10 ^∘^K), which imposes significant system-level cost and complexity, and the difficulty of scaling the sharp interface quality demonstrated on small test structures to large-format FPAs. The five-orders-of-magnitude dark current difference between sharp and gradual interfaces [107] implies that even modest degradation of interface abruptness during large-area epitaxial growth could significantly compromise array uniformity. The 16 × 16 format demonstrated to date [108] remains far below the megapixel scales routinely achieved in HgCdTe or type-II superlattice VLWIR FPAs, and the scaling path has not yet been validated. Furthermore, the doping uniformity of the Si:P active layer over large areas—and the associated pixel-to-pixel dark current variation—has not been characterized at the level required for astronomical imaging applications, where sub-percent non-uniformity is typically demanded.

## 8. Single-Photon Avalanche Diodes (SPADs)

In Geiger-mode operation, a single avalanche event—whether triggered by a real photon or by a thermally generated carrier—produces an identical macroscopic output pulse. Dark count rate (DCR) thus plays the role that dark current plays in linear photodiodes, and suppressing it is equally critical for quantum communication, single-photon LiDAR, and fluorescence lifetime imaging [4,109,110].

### 8.1. Silicon SPAD Dark Count Reduction

Silicon benefits from a mature CMOS process base, and silicon SPADs can achieve respectable dark count rates at room temperature without exotic processing [4,111,112]. Guard ring engineering is still critical: premature edge breakdown and TAT-driven dark counts at STI corners are persistent problems, and the guard ring geometry needs to be codesigned with the junction depth [4,109].

A deep-junction SPAD incorporating a field polysilicon gate structure above the high-voltage n-well achieved 76.6% suppression of dark count noise, apparently by reducing the localized field enhancement at STI sidewalls [113]. Virtual epitaxial guard rings—formed by selective doping rather than physical trenches—specifically target STI-interface trap-driven dark counts and have demonstrated meaningful DCR reduction [114]. On the higher-performance end, backside-illuminated SPADs with doping-compensated avalanche regions have reached photon detection efficiencies above 84%, while waveguide-coupled silicon SPADs operating at room temperature show single-photon detection efficiencies above 6% for visible wavelengths [115]. Active quenching circuit optimization, by more cleanly defining the deadtime and suppressing afterpulsing, can reduce effective DCR by over 35-fold without any change to the detector itself [116].

### 8.2. InGaAs/InP SPAD Dark Current Mechanisms

InGaAs/InP SPADs are the predominant detectors for single-photon counting at 1.3–1.55 μm telecommunications wavelengths, but their narrower bandgap means that dark count rates are intrinsically higher and more sensitive to temperature than in silicon [7,79,117]. The separate absorption, grading, charge, and multiplication (SAGCM) layer structure is the standard architecture: by confining absorption to InGaAs and multiplication to InP, it independently optimizes both efficiency and noise [96].

Guard ring optimization in these devices is especially consequential. The AGR depth tuning described earlier—targeting the breakdown locus crossover point—directly determines whether the device operates in a guard-ring-limited or active-area-limited regime [64]. Combined AGR + FGR implementations push breakdown voltage up by approximately 1.5 V while maintaining peripheral dark counts within acceptable levels [64].

Afterpulsing—where carriers trapped during avalanche are released in subsequent detection windows and register as false counts—is an additional noise source peculiar to Geiger-mode operation. High-frequency gating and negative-feedback quenching circuits both address this by shortening the avalanche duration and carrier injection [7,109,118]. With optimized SAGCM design and gating, InGaAsP/InP SPADs have reached sub-kHz dark count rates while maintaining 20% photon detection efficiency [119].

## 9. CMOS Image Sensor Dark Current Reduction

CMOS image sensors operate under constraints that set them apart from discrete photodiodes: millions of pixels must meet uniform noise specifications, each pixel is surrounded by STI-bounded transistors that introduce their own interface states, and thermal budgets are tightly constrained by back-end metals [5,6,120]. Dark current management in these devices consequently draws on a different mix of techniques than those used for individual detectors.

### 9.1. Process Optimization

Forming gas anneal (FGA) at the end of device processing passivates $P_{b}$-center dangling bonds at the Si/SiO_2_ interface by driving atomic hydrogen into the oxide—a step that can meaningfully reduce mean dark current [46]. The risk is that subsequent high-temperature or high-energy plasma steps during back-end integration can reverse this passivation by dissociating Si-H bonds. Controlling plasma parameters in metal deposition, etch, and hard-mask steps is, therefore, nearly as important as the FGA itself [86]. Low-temperature back-end sequences generally preserve passivation better than conventional flows [75].

STI interface engineering deserves particular attention. Random telegraph signal noise and elevated dark current in advanced nodes frequently trace back to the STI corner region, where lithographic and etch damage creates a high density of generation centers [23,24,25]. P-Well and P^+^ implant geometry optimization around the STI edge has been reported to lower the mean dark signal rate by over 40 mV/s—where the dark signal rate (in mV/s) is the voltage accumulated on the pixel sense node per unit integration time, a standard proxy metric for dark current in CMOS image sensor characterization [23].

### 9.2. In-Pixel Compensation Techniques

When residual dark current cannot be eliminated by process means alone, in-pixel compensation offers a practical alternative. Embedding a small temperature sensor—exploiting the inherent temperature sensitivity of the 4T pixel’s source follower transistor—alongside each photodiode allows real-time estimation and subtraction of the local dark current over the operating range of −40 °C to 90 °C [121]. This is particularly valuable for automotive and outdoor imaging applications, where ambient temperature can vary by over 100 °C during normal operation.

For isolated hot pixels—which often result from single metallic contamination events or process-induced damage—on-chip detection and correction circuits provide a hardware complement to software-side dark frame subtraction. Machine learning methods for adaptive dark current correction and hot-pixel identification are an emerging direction that shows promise for next-generation image signal processors [5].

### 9.3. Negative Transfer Gate Bias Operation

An effective and low-cost bias technique involves applying a small negative voltage (around −0.7 V) to the transfer gate during integration rather than leaving it at ground [102]. At this bias, holes accumulate at the Si/SiO_2_ interface under the gate, effectively suppressing the generation–recombination activity of interface traps in that region. The result is a reported 83.9% reduction in dark current without any process modification [102]—an attractive option because it can often be implemented at the circuit level in existing designs.

## 10. Material-Specific Considerations

### 10.1. Silicon Photodiodes

Silicon’s technological maturity translates into an unusually rich toolkit for dark current management [51,52,103,105]. Black silicon—silicon whose surface has been structured into deep nanoscale pillars or cones—offers near-zero reflection and broad spectral sensitivity, but its high surface-to-volume ratio would normally lead to severe dark current. The combination of b-Si texturing with ALD Al_2_O_3_ passivation resolves this tension, delivering near-unity quantum efficiency while maintaining low dark current [51,54]. Metal-assisted chemical etching (MACE) combined with ALD has demonstrated similarly strong surface quality [103,105], and boron-implanted b-Si achieves essentially ideal spectral responsivity from 200 to 1000 nm [105]. At the application end, silicon photodiodes for medical imaging have been demonstrated with dark currents below 0.2 nA/cm^2^, which is sufficient for dose-sensitive radiographic applications.

### 10.2. Germanium and Ge-on-Si Photodiodes

Germanium photodiodes extend sensitivity to 1.6 μm, covering the C and L telecommunication bands, but the smaller bandgap (0.67 eV vs. 1.12 eV for Si) means intrinsic carrier concentration is approximately six orders of magnitude higher at room temperature, which directly translates into much higher dark current [3,90,122]. GeO_2_ passivation plays a role analogous to SiO_2_ on silicon but presents greater challenges: native GeO_2_ is water-soluble and thermally unstable above approximately 450 °C, so careful process control is needed to obtain a stable, low-defect interface [59,60]. With proper gas-phase doping combined with GeO_2_ passivation, bulk current densities as low as 0.032 mA/cm^2^ have been demonstrated [59].

Threading dislocations from the 4% lattice mismatch between Ge and Si are a persistent dark current source in Ge-on-Si epitaxial films. Cyclic thermal annealing has been the most widely adopted solution [89], and Ge p-i-n devices on oxygen-annealed substrates demonstrate particularly clean dark current characteristics [91,92]. Waveguide-integrated Ge detectors on CMOS photonic platforms add further demands, since process compatibility with Si photonics limits available thermal budgets and requires careful interface engineering [92,123,124].

### 10.3. InGaAs/InP Avalanche Photodiodes

InGaAs/InP APDs serve a critical role in optical communications and quantum key distribution, where the combination of high sensitivity and low noise at 1.3–1.55 μm is essential [2,93,117]. The narrower bandgap of In_0.53_Ga_0.47_As (∼0.74 eV, lattice-matched to InP) compared to silicon means that the intrinsic carrier concentration is approximately four orders of magnitude higher at room temperature, making dark current a more severe constraint and placing greater demands on both material quality and device architecture.

The dark current in InGaAs/InP APDs is governed by a delicate balance between SRH generation and tunneling. In the absorber, the moderate bandgap and residual background doping produce SRH generation rates that exceed those in silicon by orders of magnitude for comparable trap densities. In the multiplication layer, the high electric field required for impact ionization can simultaneously activate TAT and BTBT if the layer is not properly designed. The SAGCM (separate absorption, grading, charge, and multiplication) layer structure is the dominant architecture precisely because it decouples these two regimes: the electric field in the InGaAs absorber is kept low enough to suppress tunneling, while the field in the InP multiplication layer is maintained high enough for efficient impact ionization [96]. Only within a specific multiplication layer thickness window—typically 200–500 nm—does the device operate in the desirable regime where SRH generation, rather than tunneling, governs the dark current [93,96]. Below this window, tunneling dominates and dark current rises steeply; above it, the gain-bandwidth product degrades.

Geiger-mode SPADs derived from this structure have reached sub-kHz dark count rates at 20% photon detection efficiency [119]. Afterpulsing remains a challenge; high-frequency gating and negative-feedback techniques continue to push the practical operating repetition rate higher [7,109,118]. Two-step diffusion control—balancing shallow and deep zinc diffusion profiles—is critical for defining both the lateral extent of the active area and the electric field uniformity [79,111].

Surface leakage in InGaAs/InP devices presents challenges distinct from those in silicon. The InGaAs surface is chemically reactive and forms a native oxide of variable and generally poor quality, making mesa-etched devices particularly susceptible to peripheral dark current. ALD Al_2_O_3_ passivation applied to InGaAs guard-ring PIN photodiodes has achieved a 60% reduction in dark current relative to conventional SiO_2_ passivation [125], though this improvement is modest compared to what ALD achieves on silicon—a difference that reflects the more challenging surface chemistry of III–V compounds. Comparative studies of SiN_*x*_ versus polyimide mesa passivation show SiN_*x*_ achieving 2–8 times lower surface current for GaAs-based APDs [126]. Planar (non-mesa) device geometries based on selective-area diffusion avoid exposing the absorber sidewalls entirely, which is one reason why planar InGaAs/InP APDs generally exhibit lower dark current than mesa-isolated alternatives, though at the cost of more complex fabrication and larger guard ring area.

A practical consideration specific to InGaAs/InP detectors is the strong temperature sensitivity of dark current. Because the InGaAs bandgap is only 0.74 eV, the dark current approximately doubles for every 8–10 ^∘^K temperature increase near room temperature—compared to approximately every 7–8 ^∘^K for silicon. This makes thermoelectric cooling almost mandatory for low-noise telecom receivers and essential for SPAD operation, where typical operating temperatures of −30 to −50 ^∘^C are standard [7,79].

### 10.4. GeSn Alloy Photodiodes

GeSn alloys are attracting significant interest because increasing the Sn fraction progressively extends the absorption edge toward 2 μm and beyond, eventually driving an indirect-to-direct bandgap transition near 8–10 at.% Sn that could enable silicon-platform MWIR emission and detection without III–V wafer bonding [88,127]. From a dark current perspective, this material system presents a unique combination of challenges that distinguishes it from both pure Ge and III–V narrow-bandgap detectors.

The dominant dark current mechanism in GeSn photodiodes shifts with Sn composition. At low Sn fractions (<6 at.%), the dark current budget is dominated by defect-related SRH generation through threading dislocations introduced by the lattice mismatch between GeSn and Ge or Si substrates. At higher Sn concentrations, as the bandgap narrows below approximately 0.5 eV, the intrinsic carrier concentration rises rapidly and bulk diffusion current becomes competitive with defect-mediated generation [88]. This compositional crossover has important design implications: for low-Sn devices targeting the 2–2.5 μm range, defect reduction is the primary lever for dark current suppression, whereas for high-Sn devices targeting 3–5 μm, reducing the generation volume through thin-absorber designs becomes equally important.

Threading dislocation densities in GeSn epitaxial films are strongly composition-dependent, rising from $∼10^{7}$ cm^−2^ at 6 at.% Sn to >10^9^ cm^−2^ at 12 at.% Sn [88,127]. Unlike in Ge-on-Si, where cyclic thermal annealing can reduce dislocation densities by orders of magnitude, the thermal budget for GeSn is severely limited by Sn segregation and surface desorption above approximately 350–400 ^∘^C, making high-temperature annealing impractical. This thermal budget constraint also rules out most conventional passivation techniques. The most effective demonstrated mitigation is silicon surface passivation of GeSn mesa sidewalls via a thin Si overgrowth layer, which reduces surface leakage by approximately 100× while remaining compatible with CMOS processing [32,34,127]. This approach works by providing a chemically stable Si/SiO_2_ interface in place of the inherently unstable GeSn surface, effectively sidestepping the native oxide problem. However, the Si overgrowth introduces an additional heterointerface that may itself generate dark current if the growth quality is not carefully controlled, particularly at high Sn fractions where the lattice mismatch between GeSn and the Si cap increases.

For GeSn p-i-n detectors operating in the MWIR, dark current densities on the order of 0.1–1 A/cm^2^ at room temperature have been reported [88], which are several orders of magnitude higher than those of competing InSb or HgCdTe technologies at the same wavelengths. Cooling to 200 ^∘^K or below substantially reduces the SRH component but does not address the defect-density floor, and the practical operating temperature for GeSn MWIR detection remains an open question that depends on future progress in epitaxial quality.

### 10.5. Narrow-Bandgap Infrared Platforms: HgCdTe and Type-II Superlattices

While this review focuses primarily on silicon, germanium, and III–V photodiodes, a brief contextualization against HgCdTe and type-II superlattice (T2SL) platforms is warranted, as these represent the state of the art for MWIR and LWIR detection against which emerging alternatives are benchmarked. In HgCdTe, the dominant dark current mechanism at operating temperatures of 77–200 ^∘^K is typically Auger generation in the narrow-gap absorber, supplemented by SRH generation through Hg vacancies that are native point defects in this material [30]. Decades of materials optimization have reduced dark current densities in MWIR HgCdTe to values approaching the Auger-limited floor (Rule 07), but the requirement for lattice-matched CdZnTe substrates limits wafer size and increases cost [30]. Type-II InAs/GaSb superlattices offer a design alternative in which the effective bandgap is controlled by layer thicknesses rather than alloy composition, and recent progress has narrowed the dark current gap with HgCdTe, though SRH generation through interface defects remains the performance-limiting mechanism in most T2SL devices. For photon-trapping structures with thin absorbers, dark current nearly three orders of magnitude below comparable HgCdTe APDs has been demonstrated [29], illustrating the potential of architectural approaches to supplement material quality improvements.

### 10.6. 2D Van der Waals Heterostructures

In the 2D materials space, van der Waals heterostructures based on MoS_2_ and VS_2_ offer fundamentally different dark current characteristics than bulk semiconductors: the absence of dangling bonds at van der Waals interfaces removes the dominant surface generation mechanism, and the atomically thin absorber reduces the bulk generation volume [99,100]. Graphene/Si heterojunctions benefit from thin high-*k* interlayers that suppress thermionic dark current at the Schottky-like interface. More recently, van der Waals epilayers on HgCdTe have enabled fast uncooled MWIR detection by combining the advantages of both material classes [27].

An objective assessment of emerging material platforms must acknowledge that the reported dark current figures are typically obtained on individual devices fabricated under laboratory conditions, and the path to scalable production remains unclear for all of them. For GeSn, the threading dislocation density increases steeply with Sn fraction—precisely the compositional range needed for direct-bandgap mid-infrared operation—creating an intrinsic tension between spectral reach and dark current performance that growth optimization has not yet resolved [88,127]. For 2D materials, the ultra-low dark currents measured on exfoliated-flake devices reflect pristine van der Waals interfaces that are difficult to reproduce by scalable growth techniques such as CVD, where grain boundaries and transfer-related contamination introduce additional generation centers. Whether 2D heterostructure photodetectors can maintain their interface quality advantage after full CMOS process integration—including lithographic patterning, dielectric encapsulation, and metallization—has not been demonstrated at the array scale. Furthermore, the measurement conditions under which ultra-low dark currents are reported for these devices (small active areas, low bias, controlled atmospheres) may not be representative of operational environments.

**Figure 7:**
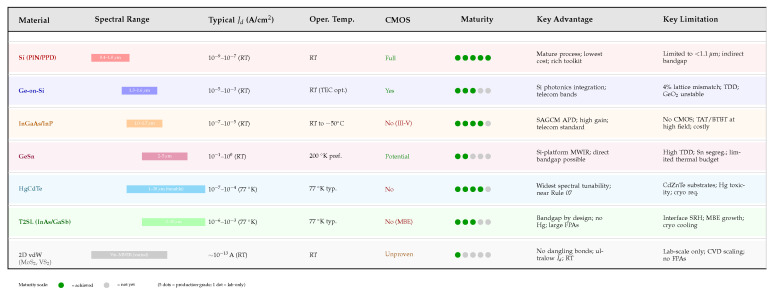
Comparative overview of material-specific photodiode platforms discussed in this review. Spectral range bars indicate approximate detection bandwidth; typical dark current densities ($J_{d}$) are representative values at the stated operating temperature; maturity is indicated on a five-level scale from laboratory demonstration (1 filled dot) to production-grade technology (5 filled dots). The rightmost column lists all references cited in this review for each material platform. Each platform presents a distinct trade-off profile: silicon offers unmatched process maturity and CMOS compatibility but is limited to the visible–near-IR; III–V and narrow-gap platforms extend spectral coverage at the cost of CMOS incompatibility and cryogenic cooling requirements; emerging platforms (GeSn, 2D materials) offer attractive spectral and dark current properties but have not yet demonstrated scalable fabrication.

Figure 7 provides a consolidated visual comparison of the key trade-offs across the material platforms discussed in this section, highlighting spectral coverage, dark current levels, operating temperature, CMOS compatibility, and technology readiness.

## 11. Literature Classification

The literature surveyed in this review is organized into four thematic classification tables (Table 1, Table 2, Table 3 and Table 4), each grouping publications by technique category and reporting both the dark current or dark count reduction achieved and, where available, additional performance metrics. To address the inherently heterogeneous nature of figures of merit across different device types, the “Outcome Metric” column explicitly labels what quantity was measured or improved—distinguishing, for example, dark current reduction ratios ($I_{d}$ red.) from absolute dark current values ($I_{d}$ abs.), dark count rate (DCR), breakdown voltage ($V_{\mathrm{br}}$), quantum efficiency (QE), photon detection probability/efficiency (PDP/PDE), and dark signal rate (DSR, in mV/s). Entries marked “Diag.” denote diagnostic or mechanistic studies without a single reduction figure, and “Qual.” denotes qualitative improvements where no numerical metric was reported. This labeling enables the reader to compare entries within and across categories without conflating fundamentally different figures of merit.

## 12. Cross-Platform Comparative Analysis

To complement the technique-by-technique classification in Table 1, Table 2, Table 3 and Table 4, Table 5 provides a platform-level comparison that maps each major photodiode material system to its dominant dark current mechanism, the most effective mitigation strategy, the reported reduction magnitude, and the key practical trade-offs that govern deployment. Table 6 then presents quantitative dark current benchmarks extracted from the literature alongside the measurement conditions (temperature, bias voltage, device area) under which they were obtained, enabling more direct numerical comparison across platforms. Together, these cross-platform views are intended to help the reader identify, at a glance, which combination of strategies is most appropriate for a given application context and to appreciate the quantitative gulf that separates different material systems under comparable operating conditions.

A persistent challenge in comparing dark current results across the literature is the inconsistency of reporting conditions. Dark current values are variously quoted as absolute currents (nA or pA), current densities normalized to junction area (A/cm^2^), dark count rates (Hz or cps), or dark signal rates (mV/s), and the operating temperature, reverse-bias voltage, and device active area differ substantially between studies. To facilitate more direct quantitative comparison, Table 6 extracts representative dark current density values from the literature and presents them alongside the measurement conditions under which they were obtained. Where the original publication reports absolute current and device area separately, we compute the current density; where only absolute current is reported without device area, the entry is marked accordingly. The reader should note that even after area normalization, direct comparison between platforms requires caution because the relative contributions of bulk generation, surface generation, and peripheral leakage scale differently with device geometry—a point that is especially relevant when comparing large-area discrete photodiodes with small-pixel imaging arrays.

**Figure 8:**
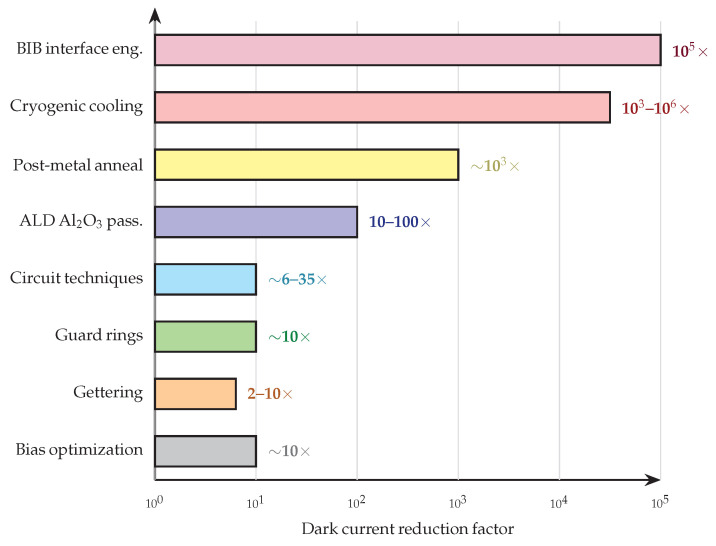
Approximate dark current reduction factors achievable by major technique categories, shown on a logarithmic scale. Values represent the best reported results from the literature surveyed; actual performance depends on the specific device, material platform, and operating conditions. The wide dynamic range (from ∼2× for basic gettering to ∼10^5^× for BIB interface engineering and ∼10^6^× for deep cryogenic cooling) underscores that the most effective approaches tend to impose the most demanding system-level constraints (e.g., 4 ^∘^K cooling for BIB, 77 ^∘^K for cryogenic). Techniques in the 10–100× range (passivation, guard rings) are generally compatible with room-temperature, CMOS-integrated operation.

The analytical observations below synthesize the cross-cutting patterns that emerge from Table 5 and Table 6, followed by an explicit discussion of how dark current reduction interacts with other key device performance parameters. Figure 8 provides a visual summary of the approximate dark current reduction magnitude achievable by each major technique category, illustrating the wide dynamic range of effectiveness and the complementary nature of different approaches.

### 12.1. Cross-Cutting Patterns Across Platforms

Several cross-cutting observations emerge from this comparison. First, the dominant dark current mechanism shifts systematically with bandgap: wide-bandgap silicon devices are governed by interface SRH generation, narrow-gap Ge and InGaAs devices increasingly contend with tunneling (TAT and BTBT), and the VLWIR Si:P BIB platform is dominated by hopping conduction through the impurity band. Second, the most effective mitigation strategies are platform-specific—no single technique is universally optimal. ALD Al_2_O_3_ passivation, for example, delivers transformative gains on silicon and black silicon but plays a more modest supplementary role on InGaAs, where guard ring and layer structure codesign are the primary levers. Third, practical deployment constraints—CMOS compatibility, operating temperature, and array scalability—often dictate the viable strategy space as strongly as the achievable dark current reduction itself. Silicon-based platforms benefit from native CMOS compatibility and room-temperature operation, whereas III–V and VLWIR BIB devices require dedicated fabrication lines and active cooling, respectively.

### 12.2. Trade-Offs Between Dark Current Reduction and Device Performance

An important dimension that cuts across all platforms is that dark current reduction rarely comes for free—nearly every mitigation strategy entails a trade-off against one or more of responsivity, noise figure, or bandwidth. Understanding these trade-offs is essential for selecting the optimal strategy for a given application.

**Responsivity and quantum efficiency.** Several of the most effective dark current reduction techniques directly compete with optical performance. Guard ring structures consume lateral chip area that would otherwise collect photons, reducing the geometric fill factor—an especially acute penalty in high-density focal plane arrays with sub-10 μm pixel pitches, where even a single floating guard ring can sacrifice 10–20% of the collection area [62,64]. Thin-absorber designs reduce the SRH generation volume and can suppress dark current by two to three orders of magnitude, but they simultaneously reduce the single-pass absorption efficiency unless compensated by photon-trapping structures. Surface passivation layers, while highly effective at suppressing surface generation, can introduce parasitic optical absorption or antireflection mismatches: ALD Al_2_O_3_ absorbs below 200 nm, and its refractive index must be matched to the detector stack to avoid reflective losses in the UV [51,52]. The optimized AGR depth in InGaAs/InP devices that yields a 70% dark current reduction also modifies the lateral carrier collection profile, with reported quantum efficiency changes of up to 43%—an improvement in that case, but one that is design-specific and can go in either direction depending on the AGR geometry [62].

**Bandwidth and speed.** Dark current reduction and high-speed operation impose conflicting requirements on the depletion region width. Widening the intrinsic layer reduces the peak electric field (suppressing TAT and BTBT) but increases carrier transit time and junction capacitance, both of which degrade bandwidth [2,94]. In InGaAs/InP APDs, the multiplication layer thickness must be optimized jointly for gain, noise, and dark current: layers thinner than approximately 200 nm enter a tunneling-dominated regime with high dark current, while layers thicker than approximately 500 nm sacrifice gain-bandwidth product [93,96]. For CMOS image sensors, the negative transfer gate bias that achieves 84% dark current reduction [102] slightly increases the charge transfer time from the photodiode to the floating diffusion, which may limit the maximum frame rate in high-speed imaging applications. Thermoelectric cooling, while universally effective at dark current suppression, can increase the carrier transit time through reduced mobility at lower temperatures in narrow-gap materials, partially offsetting the bandwidth gains from reduced RC parasitics.

**Noise figure and excess noise.** In avalanche photodiodes, the dark current reduction strategy interacts directly with the excess noise factor F(M), which depends on the ionization coefficient ratio *k* and is governed by the multiplication layer material and thickness [2]. The SAGCM architecture that separates the absorption and multiplication functions to minimize dark current simultaneously controls *k* by confining multiplication to InP (where k<1), yielding lower excess noise than would be achievable with a homojunction design—an example where dark current and noise figure optimization are synergistic rather than competing [96]. However, in SPADs, the aggressive deadtime imposed by active quenching circuits to suppress afterpulsing-related dark counts directly limits the maximum photon count rate, creating a fundamental throughput–noise trade-off [109,116]. Similarly, the forming gas anneal used to passivate interface traps in CMOS image sensors introduces hydrogen into the gate oxide, which can increase low-frequency (1/f) noise in the in-pixel transistors even as it reduces the dark current generation rate [46].

**Practical deployment constraints.** Beyond the device-level trade-offs, system-level considerations frequently dominate the strategy selection. Cryogenic cooling eliminates thermal dark current generation almost entirely but imposes size, weight, power, and cost penalties that preclude its use in many consumer and automotive applications [30]. The BIB architecture achieves five orders of magnitude dark current suppression through interface engineering but requires 4 ^∘^K operation, limiting deployment to space-borne and laboratory environments [107]. Conversely, the in-pixel temperature compensation approach for CMOS image sensors [121] provides only modest dark current correction but operates over a −40 to 90 ^∘^C range without any external cooling infrastructure, making it the only viable option for automotive vision systems despite its inferior absolute performance.

These examples underscore a central conclusion of this review: the “best” dark current reduction strategy is not the one that achieves the largest reported suppression factor but the one that achieves the required noise performance while preserving the responsivity, bandwidth, and noise characteristics demanded by the target application.

## 13. Summary and Conclusions

This review has surveyed peer-reviewed publications covering dark current mechanisms and reduction strategies across a wide range of photodiode materials and architectures. From this body of work, several recurring observations emerge—findings that hold regardless of the particular material platform or device architecture in question.

Mechanism identification is perhaps the most underappreciated step in dark current engineering. Before any reduction strategy can be optimally targeted, the dominant contribution must be identified through temperature- and bias-dependent measurements. Arrhenius plots with activation energies close to $E_{g}$ or $E_{g}/2$ cleanly separate diffusion from SRH generation; values further below point toward tunneling. Dark current spectroscopy adds resolution by resolving individual trap contributions. Skipping this diagnostic step and applying a blanket reduction approach often leads to diminishing returns.

The most significant improvements consistently come from combining techniques rather than optimizing any single one. Thermal management, surface passivation, guard ring engineering, and gettering address physically distinct mechanisms; their benefits are approximately additive. ALD Al_2_O_3_ in particular has emerged as a versatile passivation layer that performs well on silicon, black silicon, and compound semiconductor surfaces alike, and its conformal deposition capability extends its reach to nanostructured interfaces that conventional films cannot coat.

Material specificity also matters. Silicon’s well-understood oxide chemistry and mature process infrastructure put it in a uniquely favorable position. Germanium faces a more difficult surface chemistry problem—GeO_2_ is unstable under conditions that are routine for silicon—and threading dislocations from the Ge/Si lattice mismatch add an epitaxial growth challenge on top. III–V compounds require careful guard ring and multiplication layer codesign to avoid tunneling-dominated dark current, while GeSn and 2D material platforms are still at an early stage of development in terms of passivation and defect control.

Guard ring design deserves careful attention even when it appears secondary. The combined AGR+FGR approach delivers complementary benefits—active peripheral collection plus passive voltage sharing—and the design parameters (ring depth, spacing, width) interact in non-obvious ways that TCAD optimization has helped to clarify. Novel structures like the attached step guard ring and guard-ring-free selective area growth designs suggest that the design space here is not yet exhausted.

For CMOS image sensors, the pixel-level integration context introduces constraints that bulk device engineering does not face. STI interface defects, back-end process compatibility, and the impossibility of post-fabrication annealing at device temperature make process sequence design central. Hydrocarbon molecular ion gettering, negative-bias transfer gate operation, and in-pixel temperature compensation have all demonstrated substantive gains within these constraints.

Single-photon avalanche diodes add afterpulsing as a dark count contributor beyond conventional generation mechanisms. The approaches that suppress dark count rate—deep junctions, virtual epitaxial guard rings, optimized quenching circuits, high-frequency gating—are largely independent of those that improve linear photodiode dark current, reflecting the different physics of Geiger-mode operation.

In the VLWIR domain, blocked impurity band (BIB) photodetectors based on phosphorus-doped silicon have recently demonstrated that interface engineering between the active and blocking layers can suppress dark current by up to five orders of magnitude [107], while prototype 16 × 16 FPAs have achieved sub-40 fA dark current at 4 ^∘^K with broadband response extending to 32.2 μm [108]. These results confirm that the interface-controlled dark current principles discussed throughout this review extend naturally to impurity-band architectures operating under deep cryogenic conditions—a regime of particular relevance to infrared astronomy and space-based remote sensing.

Several directions remain open. Room-temperature passivation solutions for narrow-bandgap III–V and GeSn surfaces are still immature. Machine-learning-guided TCAD optimization could accelerate guard ring and doping profile design in ways that exhaustive parametric sweeps cannot. Gettering strategies compatible with the low thermal budgets of 3D-stacked image sensors need further development. Scaling BIB FPAs to larger formats while preserving the sharp interface quality that governs their dark current performance is an important challenge for next-generation VLWIR imagers. Additionally, in 2D material heterostructures, the fundamental question of whether van der Waals interfaces can maintain their interface quality advantage after full CMOS process integration remains to be answered at scale.

## Figures and Tables

**Table 1:** Literature classification: Thermal management and surface passivation techniques.

Category	Technique	Key Finding	Outcome Metric	Reported Value	Refs.
Thermal Management	Cryogenic cooling (77 ^∘^K)	Dark current to pA level	$I_{d}$ red.	10^3^–10^6^×	[35,39]
	TEC cooling	20 ^∘^K reduction for Si	$I_{d}$ red.	10–100×	[30]
	Temperature analysis	Arrhenius identifies mechanisms	Diag.	—	[11,37,38]
	SOI PIN study	Dark current varies orders of mag.	Diag.	—	[38]
	Ge-on-Si cryogenic	SRH/TAT separation at 34–334 ^∘^K	Diag.	—	[21]
	Cryo treatment	Surface modification benefits	$I_{d}$ red.	2–5×	
Surface Pass. (Silicon)	Thermal SiO_2_	Low interface state density ($∼10^{10}$ cm^−2^eV^−1^)	Qual.	Baseline reference	[33,42,47]
	ALD Al_2_O_3_	High negative charge, field effect	$I_{d}$ red.	10–100×	[40,41,48]
	Al_2_O_3_/SiO_2_ bilayer	H_2_ plasma treatment	$I_{d}$ abs.	$J_{0}$ = 0.35 fA/cm^2^	[48]
	Post-metal. anneal	Interface charge modification	$I_{d}$ red.	1000×	[21]
	PECVD SiO_2_/Si_3_N_4_	Photon-trapping structures	$I_{d}$ red.	10^4^×	[44]
	Induced junction b-Si	Near-ideal QE 250–950 nm	QE	>96% EQE	[51,52,54]
	Black Si + pass.	MACE + surface treatment	QE	Record responsivity	[103,105]
	SiPM Al_2_O_3_	Superior UV passivation	$I_{d}$ abs.	$J_{0e}$ = 8 fA/cm^2^	[55]
	MIS (SiO_2_)	Barrier height increase	$I_{\mathrm{ph}}/I_{d}$	200× ratio	
	VS_2_/SiO_2_/Si MIS	Ultra-thin insulator	$I_{d}$ abs.	∼10^−13^ A	[99]
Surface Pass. (Germanium)	GeO_2_ plasma ox.	Lower trap density than SiO_2_/Ge	$I_{d}$ red.	10×	[60]
	GeO_2_ + gas doping	Ultra-low junction leakage	$I_{d}$ abs.	0.032 mA/cm^2^	[59]
	Si passivation (GeSn)	Mesa sidewall passivation	$I_{d}$ red.	100×	[32,34,61]
	Si:C Schottky	Enhancement for MSM	Qual.	Dark suppression	
	Ge-on-SOI MSM	Metal-Ge-metal optimization	Qual.	Lower surface leak.	
	ALD SiO_2_ on Ge	Tailorable charge polarity	Qual.	Improved $\tau_{\mathrm{eff}}$	

**Table 2:** Literature classification: Guard ring structures and gettering/defect engineering techniques.

Category	Technique	Key Finding	Outcome Metric	Reported Value	Refs.
Guard Ring Structures	Attached GR (AGR)	Edge field reshaping	$I_{d}$ red.	70%	[62,64]
	Biased AGR (0.8 V)	Active peripheral collection	$I_{d}$ abs.	∼100 nA/cm^2^	[62,65]
	Floating GR (FGR)	Depletion spreading	$I_{d}$ red.	10×	[62,63]
	Multiple FGR	Breakdown voltage increase	$V_{\mathrm{br}}$	+38 V	[70,72]
	AGR + FGR combined	Both effects combined	$V_{\mathrm{br}}$	+1.5 V	[64]
	Attached step GR	Novel diffusion structure	$V_{\mathrm{br}}$	+23%	[77]
	Double-diff. planar APD	InGaAs/InP optimization	Qual.	High gain, low $I_{d}$	[64,66]
	Multi-stage gradient	E-field shaping	Qual.	Breakdown suppressed	[76]
	Guard hole structure	High-density FPA alternative	$I_{d}$ red.	60%	
	p^+^/n^+^ combined	Leakage channel restriction	Qual.	Improved isolation	[74,75]
Gettering Techniques	P backside	Metal impurity removal	$I_{d}$ red.	2–10×	[74,80,82]
	Ion implant damage	P, As, Ar implantation	Qual.	White defect elim.	[84,85]
	C implant gettering	Mo, W capture	Qual.	Effective capture	[86]
	C_3_H_6_ mol. ion	Double epitaxial wafers	$I_{d}$ red.	40% (white spots)	[75,87]
	CH_2_P mol. ion	Enhanced gettering	$I_{d}$ red.	67%	[81]
	Low-T processing	<900 ^∘^C lifetime preservation	Qual.	Quality maintained	[74,83]
	3D-stacked BSI CIS	Thin wafer gettering	$I_{d}$ red.	White spot reduction	[75,81,87]
	Intrinsic gettering	BMD formation	Qual.	Baseline	[80]

**Table 3:** Literature classification: Avalanche photodiodes and single-photon avalanche diodes (SPADs).

Category	Technique	Key Finding	Outcome Metric	Reported Value	Refs.
InGaAs/InPAPD and SPAD	SAGCM structure	Optimized mult. layer	Diag.	G-R limited regime	[96]
	Geiger-mode SPAD	Sub-kHz DCR at 20% PDE	DCR	870 Hz	[119]
	High-freq. gating	GHz rate operation	Qual.	Afterpulse suppressed	[7,117,118]
	Shallow/deep diff.	Two-step junction control	Qual.	Performance optimized	[79,111]
	ALD Al_2_O_3_ pass.	InGaAs guard-ring PIN	$I_{d}$ red.	60%	[125]
	MIM microcavity	Active size reduction	$I_{d}$ red.	Reduced (not quant.)	
	InAlAs mult.	High bandwidth, low dark	$I_{d}$ abs.	6.3 nA at 18 GHz	
	Wafer bonding InGaAs/Si	Two-step bonding	Qual.	Gain = 235	[98]
	Wet recess etching	Mult. width control	DCR	9 kHz	
	Guard-ring-free SAG	Selective area growth	PDP	33–43%	[78]
Silicon SPAD	Deep junction SPAD	P-implant/HVNW	PDP	6.8% at 905 nm	[113,114]
	Field polysilicon gate	Dark noise suppression	DCR red.	76.6%	[113]
	Virtual epi guard ring	STI trap suppression	DCR red.	Reduced	[114]
	BSI doping compensated	High PDE, low noise	PDE	>84%	
	Waveguide coupled	Room-temperature visible	PDE	>6% SPDE	[115]
	Active quenching opt.	Afterpulse suppression	DCR red.	35×	[116]

**Table 4:** Literature classification: CMOS image sensors, black silicon, device architecture, bias management, and BIB photodetectors.

Category	Technique	Key Finding	Outcome Metric	Reported Value	Refs.
CMOS Image Sensor	Forming gas anneal	Interface passivation	$I_{d}$ red.	Mean dark curr. reduced	[46]
	Plasma process opt.	Prevent pass. dissociation	Qual.	Passivation preserved	[86]
	Negative TG bias	Interface defect control	$I_{d}$ red.	83.9%	[102]
	P-Well optimization	STI interface defects	DSR	39.8 mV/s decrease	[23,25]
	In-pixel T sensor	Real-time compensation	Qual.	−40 to 90 ^∘^C range	[121]
	Hole collection PPD	Radiation hardness	Qual.	Lower degradation	
	On-chip defect corr.	Hot-pixel correction	Qual.	Real-time processing	
	STI engineering	Interface trap reduction	$I_{d}$ red.	Significant	[23,24,25]
Black Silicon	Induced junction b-Si	Near-ideal QE 250–950 nm	QE	>96% EQE	[51,54]
	B-implanted b-Si	200–1000 nm response	QE	Near-ideal resp.	[105]
	UV b-Si photodiode	Impact ionization mult.	QE	>130% EQE	[104]
	VUV b-Si	175 nm response	QE	Near-unity	[104]
	Self-biased b-Si/ITO	Broadband heterojunction	QE	∼100% EQE	[106]
Device Architecture	Pinned photodiode	Fully depleted buried channel	Qual.	Inherently low dark	[6,101]
	MIS structure	Insulator barrier insertion	$I_{\mathrm{ph}}/I_{d}$	10^7^	[99]
	Electrostatic doping	No implant damage	$I_{d}$ red.	3 decades	
	Thin absorber	Reduced generation volume	$I_{d}$ red.	10^2^–10^3^×	[29]
	Lateral heterojunc.	Interface defect avoidance	$I_{d}$ abs.	783 nA at −5 V	[97]
	Ultra-shallow junc.	Low-damage doping	Qual.	Good junction quality	
	2D materials	Van der Waals heterostructures	$I_{d}$ abs.	Ultra-low (∼fA)	[99,100]
	MWIR heterostruct.	HgCdTe with vdW epilayers	Qual.	Fast uncooled detect.	[27]
Bias Management	Zero-bias operation	No external energy	$I_{d}$ red.	Near elimination	
	Electrical over-stress	Controlled voltage stress	$I_{d}$ red.	25%	
	Negative TG bias	Channel potential control	$I_{d}$ red.	Defect suppressed	[102]
	Optimized reverse bias	Trade-off speed vs. dark	Diag.	Application dependent	[2,94]
BIB Photodetectors	Sharp interface Si:P BIB	Interface barrier controls dark curr.	$I_{d}$ red.	10^5^×; <1 pA	[107]
	Si:P BIB FPA (16 × 16)	Broadband VLWIR, 32.2 μm cutoff	$I_{d}$ abs.	<40 fA at 4 ^∘^K	[108]

**Table 5:** Cross-platform comparison of dominant dark current sources, primary mitigation strategies, and practical trade-offs across photodiode material systems and architectures.

Platform	Spectral Range	Dominant Dark Current Source	Primary Mitigation Strategy	Best Reported Reduction	Operating Temp.	CMOS Compat.	Process Complex.	Array Scalab.	Key Trade-Off
Si PIN/PPD (CIS)	400–1000 nm	SRH at Si/SiO_2_ interface; STI defects	Pinned photodiode arch. + FGA + negative TG bias	84% (neg. TG bias alone)	RT	Native	Low	Excellent	STI corner defects limit hot-pixel tail; thermal budget constrained by BEOL metals
Black Si (b-Si)	250–1000 nm (to 175 nm VUV)	Surface SRH (high surface area)	ALD Al_2_O_3_ field-effect passivation	>10^4^× vs. unpassivated	RT	Yes	Moderate (ALD)	Good	Conformal ALD is essential; passivation quality limits whether QE >100% is achievable
Si SPAD	400–950 nm	TAT at STI; afterpulsing	Deep junction + virtual epi guard ring + active quench	77% DCR (field gate); 35× (quench)	RT	Yes	Moderate	Good	Guard ring area vs. fill factor; afterpulsing limits max. count rate
Ge-on-Si	1.3–1.6 μm	Threading dislocations (SRH); surface traps	Cyclic thermal anneal + GeO_2_ passivation	32 μA/cm^2^	RT (TEC for low noise)	Moderate	Moderate	Moderate	4% lattice mismatch; GeO_2_ thermally unstable >450 ^∘^C; limited thermal budget in Si photonics
InGaAs/InP APD (linear)	1.0–1.7 μm	TAT in absorber; peripheral leakage	SAGCM layer design + AGR/FGR guard rings + ALD pass.	60% (ALD vs. SiO_2_ pass.)	RT to −40 ^∘^C	No (III–V)	High	Limited (discrete)	Mult. layer thickness governs SRH-vs.-TAT trade-off; guard ring consumes area
InGaAs/InP SPAD	1.3–1.55 μm	SRH + TAT; afterpulsing	SAGCM + high-freq. gating + neg.-feedback quench	Sub-kHz DCR at 20% PDE	−30 to −50 ^∘^C (TEC)	No (III–V)	High	Limited	Cooling mandatory for low DCR; afterpulse probability limits gate rate
GeSn	2–4 μm (MWIR)	Threading dislocations; mesa sidewall leakage	Si sidewall passivation	100×	Cooled	Partial (group IV)	High	Immature	Dislocation density rises steeply with Sn fraction; no stable native oxide exists
Si:P BIB (VLWIR)	14–40 μm	Hopping conduction; interface leakage	Sharp blocking-layer interface engineering	10^5^× (sharp vs. gradual)	4 ^∘^K (cryo)	No	Moderate (MBE)	Demo. (16 × 16)	Requires deep cryogenic cooling; FPA scaling beyond small formats is ongoing challenge
2D vdW heterostr.	Vis–MWIR	Thermionic (Schottky); minimal surface SRH	High-*k* interlayer; atomically clean interfaces	$I_{d}∼10^{-13}$ A	RT	Unproven at scale	Low (exfol.)/High (CVD)	Immature	CMOS process integration undemonstrated at scale; reproducibility of vdW interfaces

**Table 6:** Quantitative dark current benchmarks under reported measurement conditions. $J_{d}$ = dark current density; $I_{d}$ = absolute dark current; DCR = dark count rate. Where the original study reports absolute current, the device area is listed to enable density calculation. “—” indicates the parameter was not reported in the cited publication.

Platform	Device/Structure	Dark Current (as Reported)	$J_{d}$ (A/cm^2^)	Temp. (K)	Bias ($V_{R}$)	Active Area	Key Condition	Ref.
Si PIN (PQED)	Predictable QE detector	∼1 nA (RT); ∼1 pA (77 ^∘^K)	∼10^−7^ (RT)	300; 77	−5 V	∼1 cm^2^	Calibration-grade	[35,39]
Si PPD (CIS)	4T pinned photodiode	39.8 mV/s DSR reduction	—	300	Internal (PPD)	Pixel: few μm^2^	Neg. TG bias (−0.7 V)	[23,102]
Black Si	ALD Al_2_O_3_ induced junction	<0.2 nA/cm^2^ (est.)	<2 × 10^−9^	300	−1 to −5 V	mm^2^-scale	>96% EQE; >10^4^× vs. unpassivated	[51,54]
Ge-on-Si	GeO_2_-passivated PIN	0.032 mA/cm^2^	3.2 × 10^−4^	300	−1 V	∼10^−4^ cm^2^	Gas-phase doped + GeO_2_ pass.	[59]
Ge-on-Si	Cyclic-annealed PIN	—	∼10^−2^–10^−1^	300	−1 V	∼10^−4^ cm^2^	TDD ∼10^7^ cm^−2^	[21,89]
InGaAs/InP APD	SAGCM (linear mode)	∼1–10 nA	∼10^−5^–10^−4^	300	0.9$V_{\mathrm{br}}$	*⌀* 25–80 μm	SRH-limited regime	[2,96]
InGaAs/InP APD	ALD-passivated PIN	60% reduction vs. SiO_2_	—	300	−5 V	—	ALD Al_2_O_3_ on guard ring	[125]
InGaAs/InP SPAD	SAGCM Geiger mode	DCR = 870 Hz	—	223 (−50 ^∘^C)	>$V_{\mathrm{br}}$ (gated)	*⌀* 25 μm	20% PDE at 1550 nm	[119]
Si SPAD	Deep junction + field gate	DCR: 76.6% reduction	—	300	>$V_{\mathrm{br}}$	*⌀* 10–20 μm	Field polysilicon gate	[113]
Si SPAD	Active quenching	DCR: 35× reduction	—	300	>$V_{\mathrm{br}}$	—	Optimized deadtime circuit	[116]
GeSn PIN	∼8–10 at.% Sn	0.1–1 A/cm^2^	10^−1^–10^0^	300	−0.5 to −1 V	∼10^−4^ cm^2^	TDD ∼10^8^–10^9^ cm^−2^	[88,127]
GeSn PIN	Si sidewall passivation	100× reduction	—	300	−1 V	Mesa: ∼10^−4^ cm^2^	vs. unpassivated mesa	[32]
Si:P BIB	Sharp interface (test)	<1 pA	—	4	2.7 V (fwd)	Single pixel	$D^{*}\;>\;10^{12}$ Jones	[107]
Si:P BIB	16 × 16 FPA	<40 fA/pixel	—	4	Operational	16 × 16 array	$\lambda_{c}$ = 32.2 μm	[108]
2D (VS_2_/Si MIS)	Van der Waals heterostruct.	∼10M^−13^N A	—	300	—	∼μm^2^	Exfoliated flake	[99]

## Data Availability

No new data were created or analyzed in this study. Data sharing is not applicable to this article.
